# Identification of essential genes and immune cell infiltration in rheumatoid arthritis by bioinformatics analysis

**DOI:** 10.1038/s41598-023-29153-3

**Published:** 2023-02-04

**Authors:** You Ao, Zhongbo Wang, Jinghua Hu, Mingguang Yao, Wei Zhang

**Affiliations:** Department of Orthopaedics, The Fifth Hospital of Harbin, Harbin, Heilongjiang People’s Republic of China

**Keywords:** Data mining, Databases, Functional clustering, High-throughput screening, Microarrays, Network topology, Bioinformatics

## Abstract

Rheumatoid arthritis (RA) is a common autoimmune disease that can lead to severe joint damage and disability. And early diagnosis and treatment of RA can avert or substantially slow the progression of joint damage in up to 90% of patients, thereby preventing irreversible disability. Previous research indicated that 50% of the risk for the development of RA is attributable to genetic factors, but the pathogenesis is not well understood. Thus, it is urgent to identify biomarkers to arrest RA before joints are irreversibly damaged. Here, we first use the Robust Rank Aggregation method (RRA) to identify the differentially expressed genes (DEGs) between RA and normal samples by integrating four public RA patients’ mRNA expression data. Subsequently, these DEGs were used as the input for the weighted gene co-expression network analysis (WGCNA) approach to identify RA-related modules. The function enrichment analysis suggested that the RA-related modules were significantly enriched in immune-related actions. Then the hub genes were defined as the candidate genes. Our analysis showed that the expression levels of candidate genes were significantly associated with the RA immune microenvironment. And the results indicated that the expression of the candidate genes can use as predictors for RA. We hope that our method can provide a more convenient approach for the early diagnosis of RA.

## Introduction

Rheumatoid arthritis (RA) is an autoimmune disease, that typically causes damage to the synovial membrane, cartilage, and bone and includes a wide range of symptoms, such as painful and swollen joints^1^. Besides intra-articular symptoms, RA may also be accompanied by other extra-articular symptoms, including those of cardiovascular, pulmonary, metabolic, and musculoskeletal diseases^2^. In the past decade, with the advent of novel therapeutics, the introduction of early therapy, the development of new classification criteria, and the application of new effective treatment strategies, the therapeutic revolution has dramatically improved articular and systemic outcomes^3^. Targeted therapies such as Methotrexate and biological reagents are important as first-line RA medications^1^. These therapies, coupled with emerging bioengineering and cell therapy result in a diverse group of multi-drug treatment regimens^4^. Despite advances in targeted biological and pharmacologic interventions that have recently come to market, many patients with RA continue to have inadequate responses to therapies, or intolerable side effects, with the resultant progression of their disease^5^. Considering many patients still suffer from uncontrolled symptoms, it is an urgent need for us to understand the pathogenesis and develop more precise biomarkers for early diagnosis.

Recently, high-throughput sequencing technologies have been used for whole-genome sequencing (WGS) in synovial tissues or peripheral blood from RA patients, which facilitates disease pathobiology research and novel biomarker identification^6^. Researchers suggest that early diagnosis and treatment of RA can avert or substantially slow the progression of joint damage in up to 90% of patients, thereby preventing irreversible disability^7^. Previous research indicated that 60% of the risk for the development of RA is attributable to genetic factors^8^. Thus, better utilization of these high-throughput sequencing data may provide us with a basis for an in-depth understanding of pathogenesis. In terms of the pathogenesis of RA, the uncontrolled hyperplasia of synovial fibroblasts and macrophages play a vital role in the destruction of cartilage and bone^9,10^. Pro-inflammatory cytokines secreted by fibroblasts and infiltrated immune cells can gradually cause cartilage degeneration^11,12^. Since RA is an autoimmune disease, it is necessary to further explore the immune infiltration of the samples. Recently, there have been several algorithms that can be used to infer the patient’s immune microenvironment using mRNA expression data, which may also provide a new perspective for us to have a deeper understanding of the pathogenesis of RA.

Here, we first use the Robust Rank Aggregation method (RRA) to identify the differentially expressed genes (DEGs) between RA and normal samples by integrating four public RA patients mRNA expression data. Subsequently, these DEGs were used as the input for the weighted gene co-expression network analysis (WGCNA) approach to identify RA-related modules. The function enrichment analysis suggested that the RA-related modules were significantly enriched in immune-related actions. Then the hub genes were defined as the candidate genes. Our analysis showed that the expression levels of candidate genes were significantly associated with the RA immune microenvironment and could be used as predictors for RA. We hope that our method can provide a more convenient approach for clinical RA early diagnosis.

## Methods

### Data source

The gene expression datasets for RA were retrieved from the Gene Expression Omnibus (GEO) database (https://www.ncbi.nlm.nih.gov/). The study type was restricted to expression profiling by array and human species. We used the following words to search datasets: “rheumatoid arthritis,” “RA,” “Homo sapiens,” “Microarray,” and “high-throughput sequencing”. The following inclusion criteria were used: (1) the mRNA profiling of RA patients and controls involved in the datasets was detected by a high-throughput array or next-generation sequencing. (2) datasets containing more than ten RA specimens and healthy controls, respectively; (3) total RNA was extracted from human synovial tissues of the knee joint. As a result, five GEO datasets (GSE12021, GSE55235, GSE55457, GSE77298, and GSE89408) were selected. Then the gene expression profiles and corresponding clinical annotation information were downloaded for further analysis. The detailed information on the datasets used in our study was listed in Table 1.

**Table 1:** Summary of the RA datasets involved in our study.

GSE accession	Source types	Platform	Analysis type	Total number	Control	RA
GSE77298	Synovium	GPL570	Array	23	7	16
GSE55457	Synovium	GPL96	Array	23	10	13
GSE55235	Synovium	GPL96	Array	20	10	10
GSE12021	Synovium	GPL96	Array	21	9	12
GSE89408	Synovium	GPL11154	RNA-seq	180	28	152

### Differential gene expression analysis

Previous research indicated that 60% of the risk for the development of RA is attributable to genetic factors^8^. To explore the mechanism of RA, differential expression analysis was performed between RA and control patients. Robust rank aggregation (RRA) was used to integrate the four datasets before differentially expressed genes (DEGs) identification using the “RobustRankAggreg” package in R (version 3.6.3; https://www.r-project.org/). Significant probability values obtained were analyzed for multiple testing using Bonferroni correction (p.adj). Then the fold change (FC) value was also calculated. The genes with adjust P value (p.adj) < 0.05 and |log2 (FC)| > 1 were identified as DEGs between RA and control patients, including up-regulated significant DEGs and down-regulated significant DEGs.

### Functional enrichment analysis and protein–protein interaction analysis

To explore the possible biological functions involved in DEGs, the Gene Ontology (GO) functional analysis and Kyoto Encyclopedia of Genes and Genomes (KEGG) pathway enrichment analysis were performed using the “clusterProfiler” package in R software^13,14^. The GO classification includes biological process (GO-BP), cell component (GO-CC), and molecular function (GO-MF). As for both GO and pathway enrichment analyses, a P value < 0.05 was considered as statistically significant, respectively. To further screen the possible hub genes or proteins that might be critical in the progression of RA, a PPI network was generated by the GeNets website, a web platform for network-based analysis of genetic data (http://apps.broadinstitute.org/genets). The hub genes were determined from the network by using the cytoHubba plugin with Cytoscape software (version 3.8.2).

### The weighted gene co-expression network analysis

Due to the heterogeneous and varying disease course, RA classification is critical for the clinical management of patients. Although identification of DEGs is necessary, determining their interconnection is also important. Correlation networks are increasingly being used in bioinformatics applications, with weighted co-expression networks (WGCNA) commonly used to describe the molecular mechanism and reconstruct co-expression networks of genes in different samples. The “WGCNA” package in R was used for gene co-expression network analysis. As input data, four RA expression profiles were first integrated into one using the “combat” function in the “sva” package. Then the DEGs between RA and control patients were identified using the “limma” package in R, and the genes with p < 0.05 and |log FC| > 0.25 were considered to be DEGs. The expression of DEGs was used as the input data for the WGCNA. A power of β = 4, a scale-free R2 = 0.85, a cut height of 0.25, and a minimal module size of 50, were selected as soft-threshold parameters to ensure a signed scale-free co-expression gene network. Finally, a total of 12 modules were generated. Among these modules, the blue module was highly correlated with clinical traits. We further set gene significance (GS) > 0.5 and module membership (MM) > 0.8 to define the candidate genes in WGCNA.

### Immune microenvironment infiltration in RA

Increasing evidence showed that, the uncontrolled hyperplasia of synovial fibroblasts and macrophages plays a vital role in the destruction of cartilage and bone in terms of the pathogenesis of RA^9,10^. And pro-inflammatory cytokines secreted by fibroblasts and infiltrated immune cells can gradually cause cartilage degeneration^11,12^. Infiltrating stromal and immune cells form the major fraction of normal cells in tumour tissue and not only perturb the tumour signal in molecular studies but also have an important role in cancer biology. Thus, ESTIMATE (Estimation of Stromal and Immune cells in MAlignant Tumor tissues using Expression data) was first performed to estimate tumor purity and the presence of infiltrating stromal cells/immune cells in tumor tissues utilizing gene expression according to the enrichment analysis of single sample gene set^15^. Next, we further explore the immune microenvironment infiltration in RA patients. Immune infiltration analysis by single-sample GSEA (ssGSEA) was used to evaluate 24 types of immune cells that may infiltrate into the RA immune microenvironment^16^. Then the ssGSEA scores, which represent the abundance of immune cell infiltration, were calculated using the “GSVA” Bioconductor package. And the Wilcoxon rank-sum test was applied to compare the immune infiltration between RA and control patients. To investigate the relationship between the expression of hub genes and immune cell infiltration, the Spearman’s correlation analysis was performed. Furthermore, to investigate the predictive ability of the hub genes, the receiver operating characteristic (ROC) curve analysis was performed in the training and validation cohorts. To further validate the predictive value of the hub genes, another external validation cohort was downloaded, and ROC curve analysis was performed.

### Statistical analysis

All statistical analysis was conducted with R (version 3.6.3) (https://cran.r-project.org/bin/windows/base/old/3.6.3/). The following packages were employed: clusterProfiler, limma, GSVA, WGCNA, pROC, sva. P < 0.05 was considered to indicate a statistically significant difference.

## Results

### Rheumatoid arthritis microarray datasets

We use network analysis to discover effective biomarkers for RA diagnosis. A detailed flowchart of our study is shown in Supplementary Fig. S1. According to the eligibility criteria, five high-throughput datasets of RA were obtained from the GEO database in the present study. The detailed information on the datasets used in our study was listed in Table 1. The five cohorts include: (1) GSE77298, consisting of 16 RA patients and 7 controls; (2) GSE55457, consisting of 13 RA patients and 10 controls; (3) GSE55235, consisting of 10 RA patients and 10 controls; (4) GSE12021, consisting of 12 RA patients and 9 controls; (5) GSE89408, consisting of 152 RA patients and 28 controls. There were a total of 203 RA patients and 64 controls involved in our study. Considering the number of samples in the prior four datasets was small, thus the four datasets were integrated as the training set, and the GSE89408 was used as the validation set.

### Identification of the robust differentially expressed genes

To explore the mechanism of RA, differential expression analysis was performed between RA and control patients. The RRA analysis showed that there were a total of 184 DEGs identified between RA and control patients, including 136 up-regulated genes and 48 down-regulated genes (Supplementary Table S1). Among the up-regulated genes, CXCL13 was the most differently expressed up-regulated gene (p = 2.37E−14, adjusted p = 5.12E−10), followed by IGLV1-44 (p = 7.49E−14, adjusted p = 1.62E−09). Otherwise, FOSB (p = 1.27E−11, adjusted p = 2.74E−07) and FKBP5 (p = 8.10E−10, adjusted p = 1.75E−05) were the most differently expressed down-regulated genes in the RRA analysis. The top 20 up-regulated and down-regulated genes in RA were illustrated by a heatmap (Supplementary Fig. S2A).

### Functional enrichment analysis of differentially expressed genes

**Figure 1:**
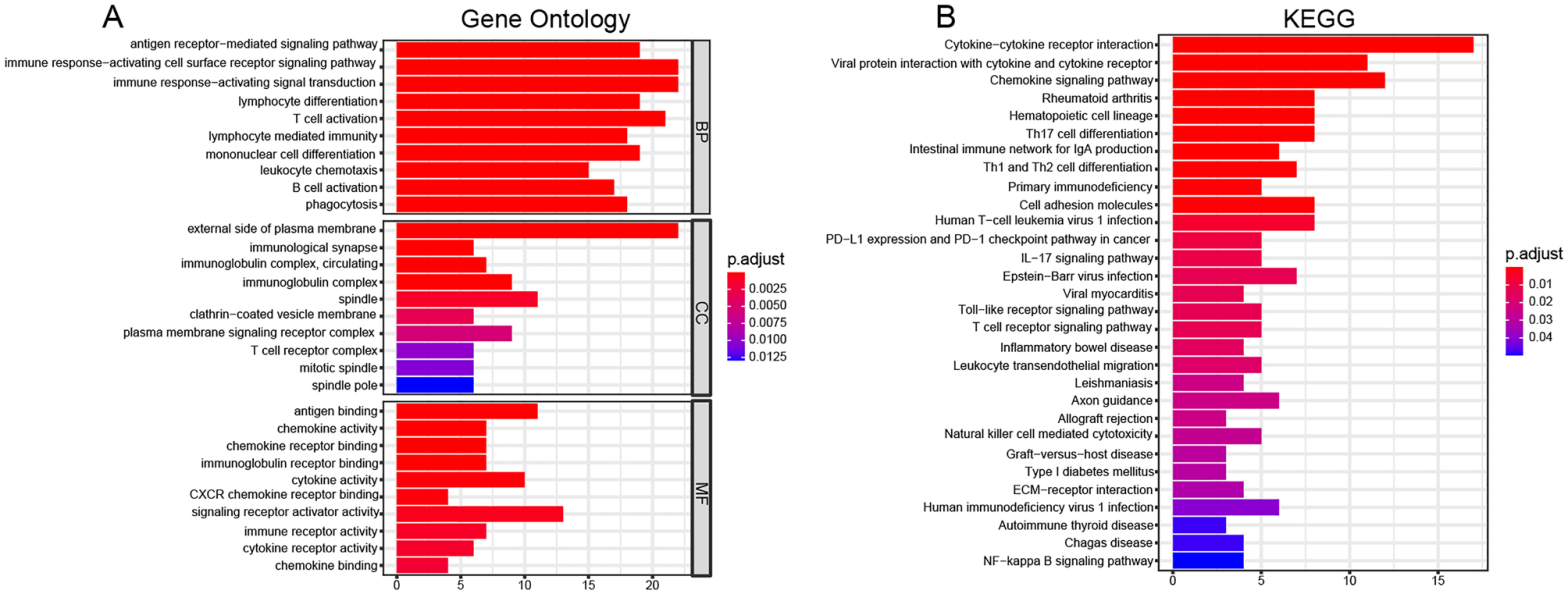
Function enrichment analysis. (**A**) GO functional analysis of the DEGs between RA and control. (**B**) KEGG pathway analysis of the DEGs between RA and control.

To explore the possible biological functions involved in DEGs, the Gene Ontology (GO) functional analysis and the Kyoto Encyclopedia of Genes and Genomes (KEGG) pathway enrichment analysis were performed. To put the DEGs into a biological context, significant enriched GO terms were identified according to their P value and enrichment factor (Supplementary Table S2). The top 10 significant enriched GO terms are summarized in Fig. 1A. As for BP, the DEGs were significantly enriched in the processes of the immune response, including antigen receptor-mediated signaling pathway, immune response-activating cell surface receptor signaling pathway, immune response-activating signal transduction, T cell activation, and B cell activation. In the CC category, DEGs were also significantly enriched in immune-related CC terms, such as external side of plasma membrane, immunological synapse, immunoglobulin complex, and T cell receptor complex. As expected, DEGs were also significantly enriched in immune-related MF terms, including antigen binding, chemokine activity, immunoglobulin receptor binding, and immune receptor activity. Moreover, the KEGG pathway enrichment analysis also indicated that the DEGs were significantly enriched in immune-related pathways, for example, Th17 cell differentiation, Th1 and Th2 cell differentiation, primary immunodeficiency, IL-17 signaling pathway, and T cell receptor signaling pathway (Fig. 1B and Supplementary Table S3). The enrichment analysis suggested that the DEGs play an important role in immune response-related biological functions.

Next, we performed a network analysis of DEGs using the GeNets. GeNets defines “communities” as sets of genes with a high degree of connectivity based on a random forest classifier trained on datasets of established protein–protein interactions. The network analysis clustered DEGs into three distinct communities (Supplementary Fig. S2B). We found that the most differently up-regulated gene, CXCL13, was also identified in the communities, highlighting that the gene with a greater different expression and it may play a vital role in the occurrence and development of the disease. The greater the node degree, the more edges this node has, and therefore could exert greater influence on the network, indicating the more biological functions they participated in. Thus, the top 20 genes based on the degree centrality included CCR5, LCK, CCL5, CXCR3, CXCL13, SDC1, ITGA4, SPP1, MMP3, ADAM28, DDX3Y, ACACB, EGR1, CD247, TNFSF11, IGHM, ITGB2, LDB3, RRM2, MS4A1, which were defined as candidate genes for further analysis.

## Weighted gene co-expression network analysis

**Figure 2:**
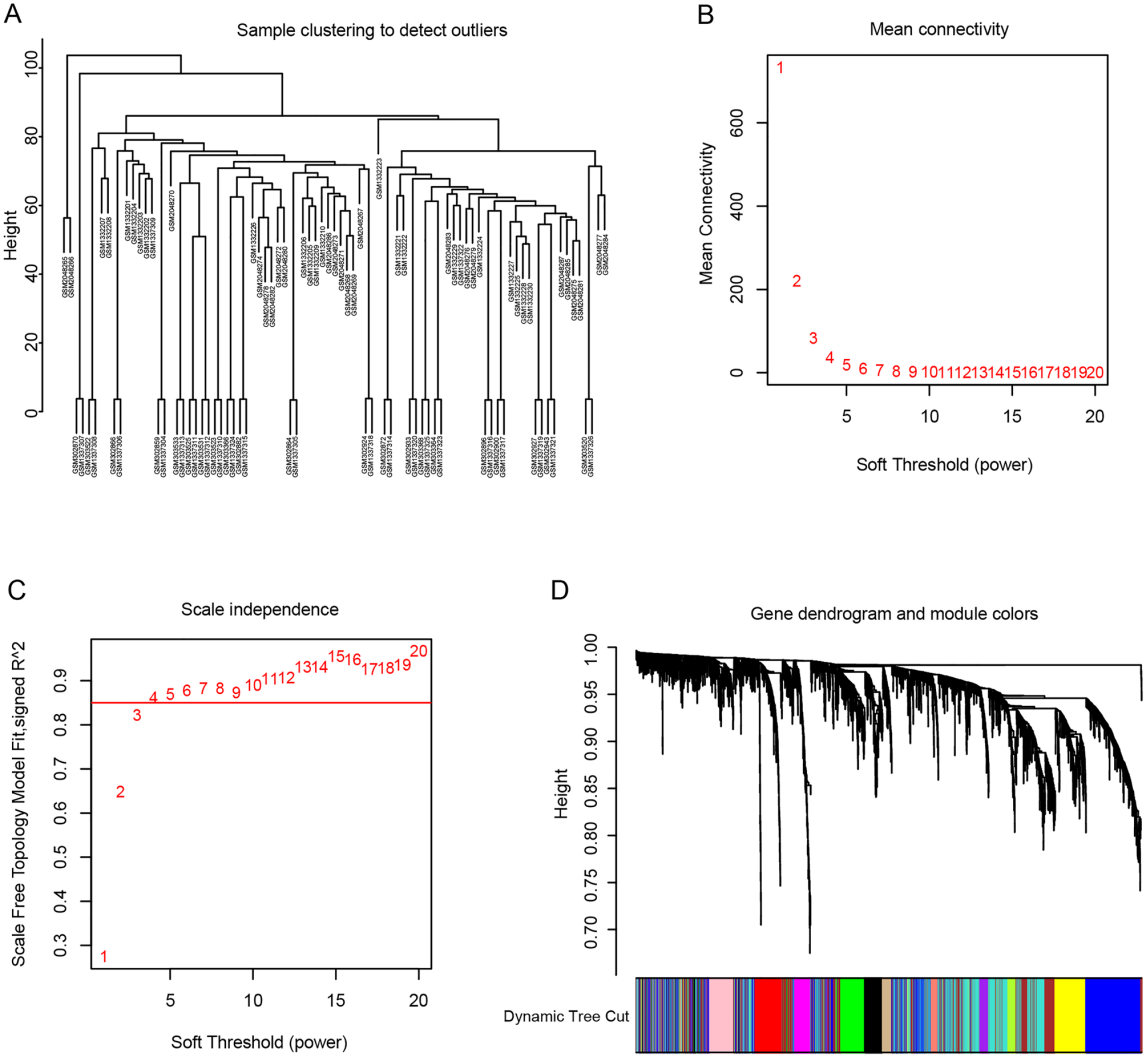
Adjacency function parameters and construction of WGCNA modules. (**A**) Sample clustering to detect outliers. (**B**) The mean connectivity of eigengenes. (**C**) Scale independence of eigengenes. The red line represents the correlation coefficient square (r2) and mean connectivity of eigengenes under a soft threshold power of 4. (**D**) Cluster dendrogram of the DEGs. Highly similar modules are identified by clustering and then merged dynamically.

**Figure 3:**
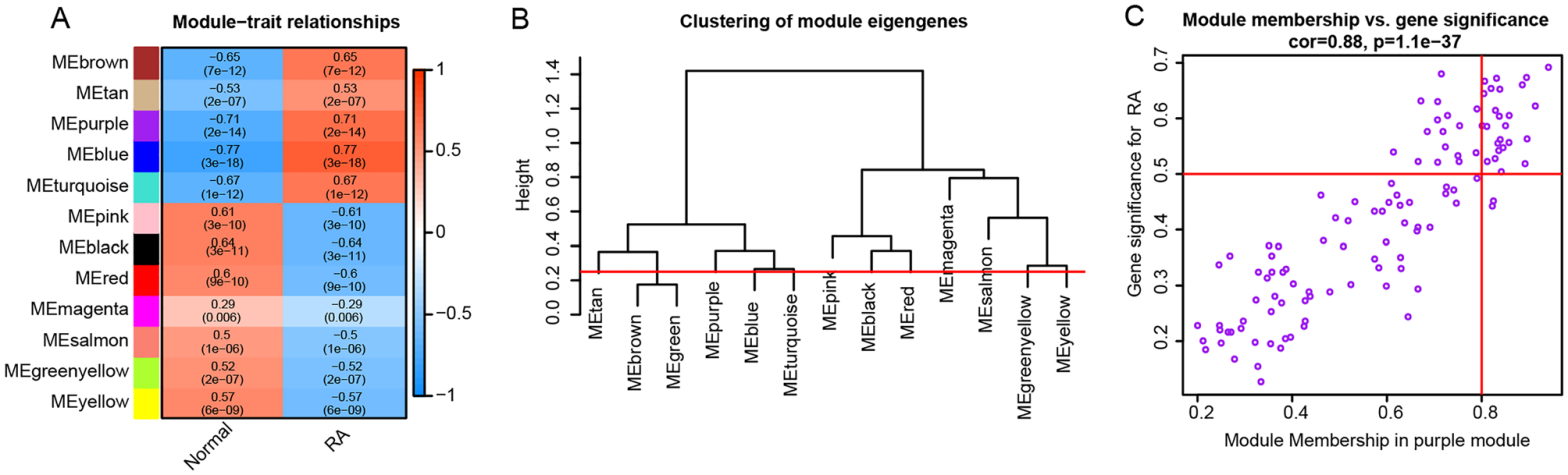
WGCNA modules analysis. (**A**) Heatmap of the WGCNA module correlations with RA and normal clinical traits. (**B**) Cluster dendrogram of the module eigengenes. The dissimilarity of module eigengenes is calculated to merge some similar modules with a height cut-off value of 0.25. (**C**) Scatter plot of the gene significance for the RA vs. the module membership in the purple module.

It has been reported that genes are not independent of each other, co-expressed genes may have similar biological functions, and the effect of grouping genes is relatively strong^17^. WGCNA algorithm, which has been widely used for studying network changes, can be used to identify network topologies and sub-networks (called modules), using topological overlap dissimilarity as a measure of the distance between genes. Considering the complexity of RA pathogenesis, the WGCNA algorithm was performed to identify biologically significant gene modules and to better understand genes associated with RA pathogenesis (Fig. 2). Due to the heterogeneous and varying disease course, RA classification is critical for the clinical management of patients. Although identification of DEGs is necessary, determining their interconnection is also important. Thus, the DEGs with p < 0.05 and logFCs > 0.25 were selected as the input data for WGCNA to generate the gene co-expression modules. Applying a soft-thresholding power of 4 (scale-free R2 = 0.85) (Fig. 2B,C) and cutting height as 0.25 (Fig. 3B), 12 RA-related modules were identified (Fig. 3A).

In the WGCNA algorithm, the hub module was identified by the highest correlation between the module eigengene and the clinical traits, as well as the most significant correlation between the module membership and the gene significance. Then the relationship between the modules and the clinical traits was evaluated to identify the hub modules (Supplementary Figs. S3 and S4). The result showed that the blue module was significantly associated with RA (correlation coefficient = 0.77, p = 3e−08) (Fig. 3C). Consequently, the blue module was identified as the hub module in the WGCNA network and we selected the top 20 genes based on the gene significance and module membership as the candidate genes: ADAMDEC1, TRBC1, CD27, SEL1L3, LCK, IGLL5, IGKC, IGLJ3, CD3D, CXCL13, TNFRSF17, IGHM, CD2, MS4A1, TRAF3IP3, HLA-DOB, IGLV1-44, TRAC, TPD52, and PSMB9 (Supplementary Fig. S2C).

### Identification of hub genes and diagnostic efficacy verification

Then the top 20 candidate genes in the PPI and WGCNA networks were overlapped to identify as the common hub genes, including LCK, CXCL13, IGHM, and MS4A1 (Supplementary Fig. S2C).

**Figure 4:**
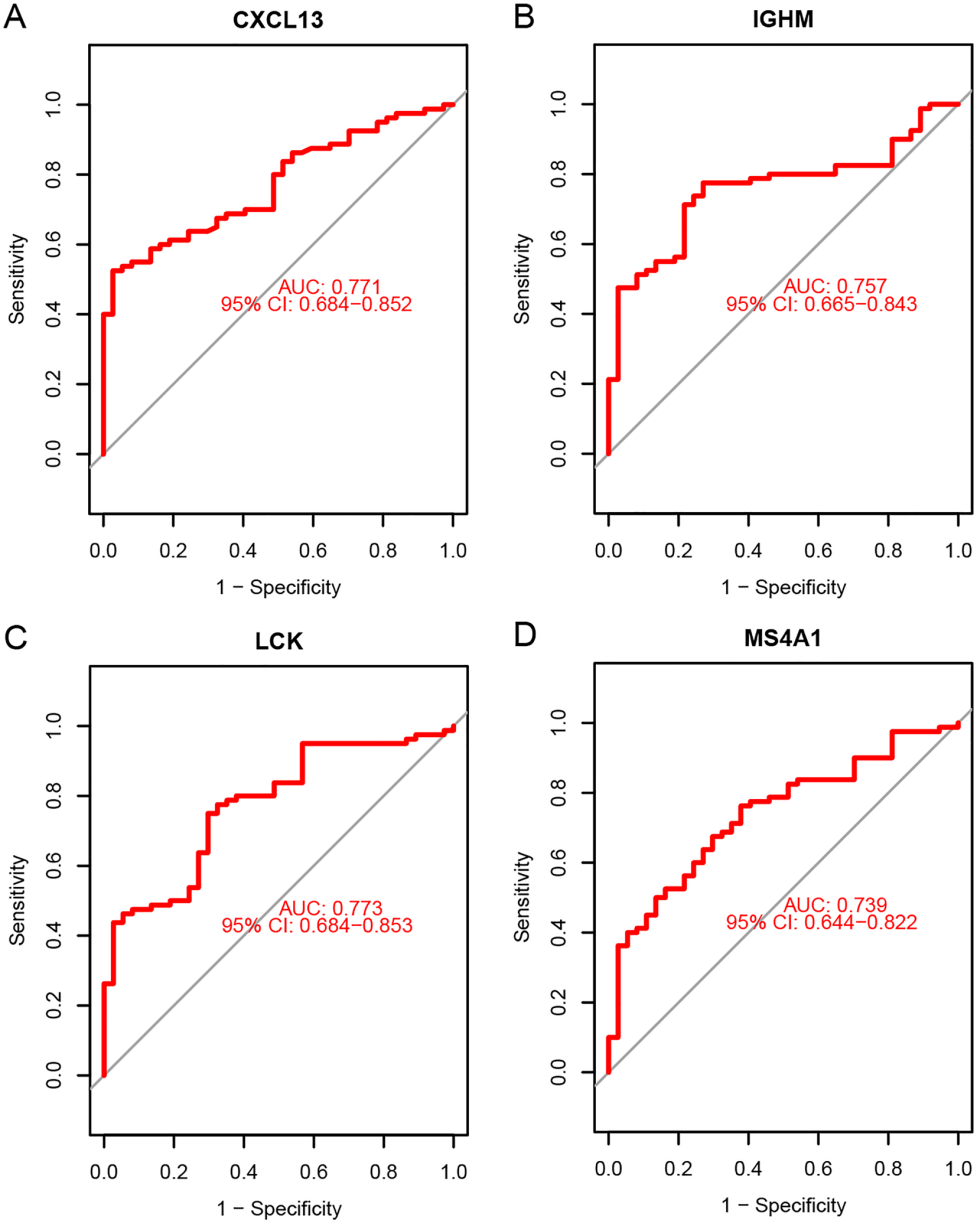
ROC curve analysis in the training cohort. (**A**) ROC curves of the expression of CXCL13 to predict RA. (**B**) ROC curves of the expression of IGHM to predict RA. (**C**) ROC curves of the expression of LCK to predict RA. (**D**) ROC curves of the expression of MS4A1 to predict RA.

Then receiver operating characteristic (ROC) curve analysis was performed to evaluate the individual predictive power of the four common hub genes. The results show that the LCK has the highest AUC value (AUC = 0.773), followed by CXCL13 (AUC = 0.771), IGHM (AUC = 0.757), and MS4A1 (AUC = 0.739) (Fig. 4), suggesting that the four common hub genes have better predictive abilities.

**Figure 5:**
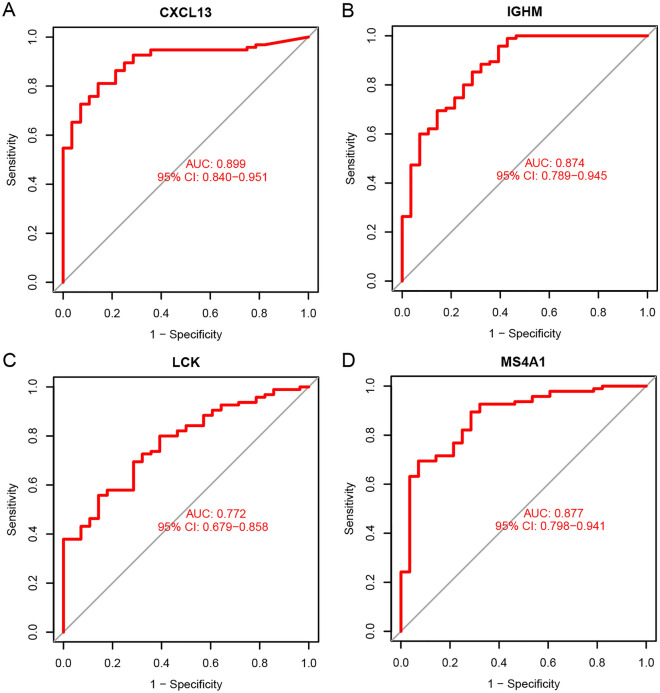
ROC curve analysis in the validation cohort. (**A**) ROC curves of the expression of CXCL13 to predict RA. (**B**) ROC curves of the expression of IGHM to predict RA. (**C**) ROC curves of the expression of LCK to predict RA. (**D**) ROC curves of the expression of MS4A1 to predict RA.

To further validate the predictive ability of the common hub genes, the ROC curve analysis was performed in the GSE89408 dataset. The results showed that the four common hub genes also existed good performance (CXCL13: AUC = 0.899; IGHM: AUC = 0.874; LCK: AUC = 0.772; MS4A1: AUC = 0.877; Fig. 5). Moreover, another rheumatoid arthritis external validation cohort was obtained from GEO database to further validate the predictive power of four common hub genes. In the external validation cohort (GSE121894), which consisted of 36 rheumatoid arthritis and 22 control patients, the ROC curve analysis showed that the four common hub genes possess a good predictive ability (CXCL13: AUC = 0.789; IGHM: AUC = 0.726; LCK: AUC = 0.622; MS4A1: AUC = 0.652; Supplementary Fig. S5). These findings indicated that the four common hub genes may serve as effective biomarkers for RA diagnosis.

### Correlation between the biomarker genes and immune-related features

**Figure 6:**
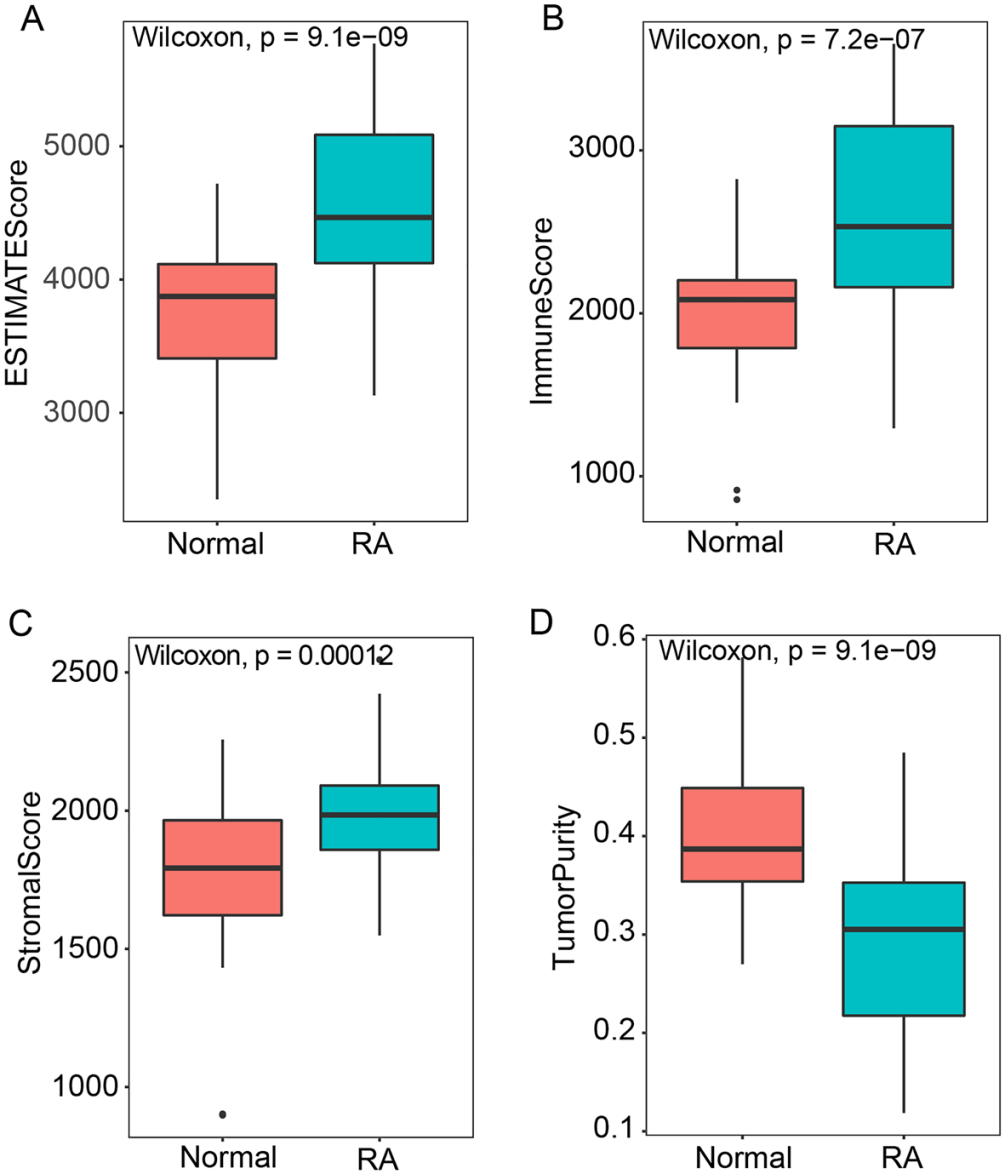
ESTIMATE analysis was performed between RA and controls. (**A**) Comparison of the ESTIMATE sore between RA and controls. (**B**) Comparison of the immune sore between RA and controls. (**C**) Comparison of the stromal sore between RA and controls. (**D**) Comparison of the tumor purity between RA and controls.

**Figure 7:**
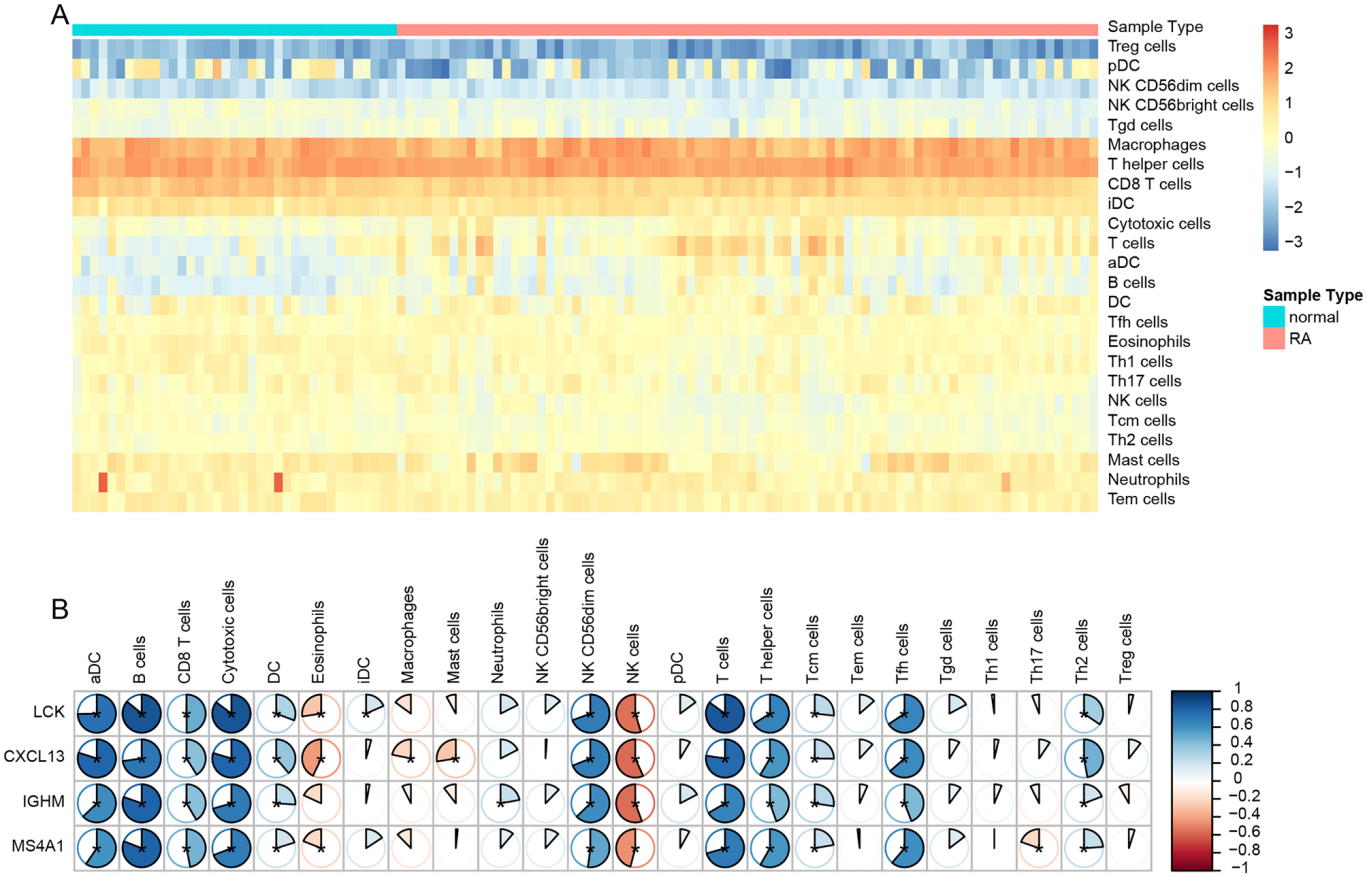
Correlation between the biomarker genes and immune infiltration. (**A**) Heatmap of the infiltration of 24 immune cells between the RA and controls drawn by the “pheatmap” package in R (version 3.6.3) (http://cran.r-project.org/bin/windows/base/old/3.6.3/). (**B**) Correlation between the biomarker genes and 24 immune cells infiltration.

Previous studies indicated that RA is an autoimmune disease, and the pro-inflammatory cytokines secreted by fibroblasts and infiltrated immune cells can gradually cause cartilage degeneration^9,10^. Therefore, exploring the correlation between the biomarker genes and immune-related features is important for our further understanding of the pathogenesis of RA. Then, we evaluated the immune state between RA and control samples with immune score and stromal score to estimate infiltrating overall immune cells and stromal cells in patients, which were calculated by the ESTIMATE algorithm. The results showed that the RA patients have significantly higher immune and stromal scores than that of control cases, as well as the ESTIMATE score (Fig. 6). The ESTIMATE results proved that there was indeed a large difference in immune microenvironment between RA samples and control samples. To further investigate which immune cells are responsible for the difference and the potential mechanism of RA. We estimated the infiltration of 24 immune cells using the ssGSEA method via the “GSVA” package in R. Compared with control samples, the RA samples had significantly higher infiltration of T cells, aDC, and B cells (Fig. 7A). Moreover, the Pearson correlation analysis was performed and the results indicated that there were 11 immune cells positively associated with the expression of four biomarker genes, including aDC, B cells, CD8 T cells, Cytotoxic cells, DC, NK CD56 bright cells, T cells, T helper cells, Tcm cells, Tfh cells, and Th2 cells. Conversely, the infiltration of NK cells was negatively associated with the expression of target genes (Fig. 7B).

## Conclusion

In summary, our study integrated relatively large sample size data to uncover the transcriptomic diversities between normal and RA synovial tissue and identified 4 essential genes (LCK, MS4A1, CXCL13, IGHM) by bioinformatics analysis. Moreover, the findings in the present research may provide deepening insights into the pathogenesis of RA and explore useful immunotherapeutic targets of RA susceptibility.

## Discussion

Rheumatoid arthritis (RA) is an autoimmune disease of the joints, that is characterized by the destruction of cartilage and bone^9^. Despite the advances in the treatment of RA, the clinical course of this disorder is marked by a continuous pattern of relapse and remission^3^. In recent years, rapid advances in bioinformatics have enabled us to screen out novel biomarkers. Therefore, identifying more novel immune-related biomarkers that could be vital in the diagnosis, treatment, and prognosis of RA is urgent.

In our study, we first used the Robust Rank Aggregation method (RRA) to identify the differentially expressed genes (DEGs) between RA and normal samples by integrating four public RA patients’ mRNA expression data. Subsequently, these DEGs were used as the input for the WGCNA approach to identify RA-related modules. The function enrichment analysis suggested that the RA-related modules were significantly enriched in immune-related functions. Then the four hub genes were defined as the biomarker genes (LCK, MS4A1, CXCL13, IGHM), which possess a good predictive ability of RA. Furthermore, our analysis also found that the expression levels of candidate genes were significantly associated with the RA immune microenvironment. The exploration of potential biomarker genes of RA may help to identify novel therapeutic targets of rheumatoid arthritis susceptibility. We hope that our method can provide a more convenient approach for clinical RA early diagnosis.

Increasing studies indicated that the DEGs, such as CXCL13, IGLV1-44, FOSB and FKBP5, were associated with the development of RA. It is well-known that chemokine signaling networks with the immune microenvironment are highly versatile and context-dependent: exert both pro-tumoral and anti-tumoral activities^16^. The CXCL13, and its cognate receptor CXCR5, represent an emerging example of chemokine signaling axes, which express the ability to modulate tumor growth and progression in other ways^18^, indicating the CXCL13 may also play an important role in the development of RA. Ye et al. suggested that IGLV1-44 might play a key role in the synovial membrane of osteoarthritis^19^. FosB recruits KAT5 to potentiate the growth and metastasis of papillary thyroid cancer in a DPP4-dependent manner^20^. The researcher also suggested that higher FKBP5 promotes inflammation by strengthening the interactions of NF-κB regulatory kinases, whereas opposing FKBP5 either by genetic deletion (CRISPR/Cas9-mediated) or selective pharmacological inhibition prevented the effects on NF-κB^20^.

The results of GO and KEGG assays indicated that 184 DEGs were mainly involved in the processes of the immune response. To further explore potential diagnostic biomarkers for RA, we performed PPI analysis and screened out the top 20 genes using the stress method. Previous research suggested that most of the top 20 genes were associated with the occurrence and development of the disease. For example, Hemmatazad et al. indicated that CCR5 is a potential therapeutic target for cancer and its higher expression is associated with poor outcomes in various malignancies and cells expressing CCR5 modulate the immune response and tumor progression^21^. Studies have shown that the LCK regulates the initiation of TCR signaling, T-cell development, and T-cell homeostasis^21^.

Correlation networks are increasingly being used in bioinformatics applications, with WGCNA commonly used to describe the molecular mechanism and reconstruct co-expression networks of genes in different samples. Therefore, using network analysis may lead to the discovery of effective biomarkers for RA diagnosis. Herein, we eventually obtained four hub genes (LCK, MS4A1, CXCL13, IGHM) from the intersection of the PPI and WGCNA methods.

Increasing evidence indicated that the four common hub genes were associated with RA^22–25^. For example, Hu et al. found that the IgD-Fc-Ig fusion protein restrains the activation of T and B cells by inhibiting IgD-IgDR-Lck signaling in rheumatoid arthritis^22^. The CXCL13/CXCR5 axis facilitates endothelial progenitor cell homing and angiogenesis during rheumatoid arthritis progression^23^. And the MS4A1 has been already developed as a target for RA treatment^24,25^. These studies suggested that the common hub genes may play important roles in the pathogenesis of RA. Thus we only focused on the common hub genes, considering the strong connection between the common hub genes and the other hub genes in the PPI and WGCNA networks.

Previous studies also indicated that the basic feature of RA is the body’s immune system disorders, in which autoreactive CD4 + T cells, pathogenic B cells, M1 macrophages, inflammatory cytokines, chemokines and autoantibodies abnormally increase in the body of RA patients^26^. And the B cell depletion therapy has well proved the important role of B cells in the pathogenesis of RA^27,28^. Fathollahi et al. found that the NK cells play an important role in the pathogenesis of RA^29^. These findings suggested that the expression of biomarker genes was associated with the patient’s immune microenvironment, and thus partly explains why the biomarker genes have better predictive power.

The advantage of our study is that we used the network analysis approach to identify potential RA biomarkers, and we also comprehensively analyzed the correlation between the biomarker genes and the patients’ immune microenvironment. Considering the limitations of RA high-throughput omics data, our method should be further validated in more RA patient cohorts in the future.

## Supplementary Information

Supplementary Information 1.

Supplementary Table S2.

## Data Availability

The datasets supporting the conclusions of this article are available in the GEO (Gene Expression Omnibus) (https://www.ncbi.nlm.nih.gov/geo/) repository. [https://www.ncbi.nlm.nih.gov/geo/query/acc.cgi?acc=GSE77298/GSE55457/GSE55235/GSE12021/GSE89408].
