# Supplementary material for: Identification of essential genes and immune cell infiltration in rheumatoid arthritis by bioinformatics analysis
Source: Sci Rep. 2023 Feb 4;13:2032. doi: 10.1038/s41598-023-29153-3 (PMC9899220; doi:10.1038/s41598-023-29153-3)
Supplement: Supplementary file 1 — Supplementary Information 1. [file 41598_2023_29153_MOESM1_ESM.docx]

Supplementary Materials for

**Identification of essential genes and Immune Cell Infiltration in rheumatoid arthritis by bioinformatics analysis**

**Inventory of Supplementary Information**

Supplementary Figure S1-S5

Supplementary Table S1, and S3

**Supplementary Figure S1. The detailed flowchart of our study.**

**
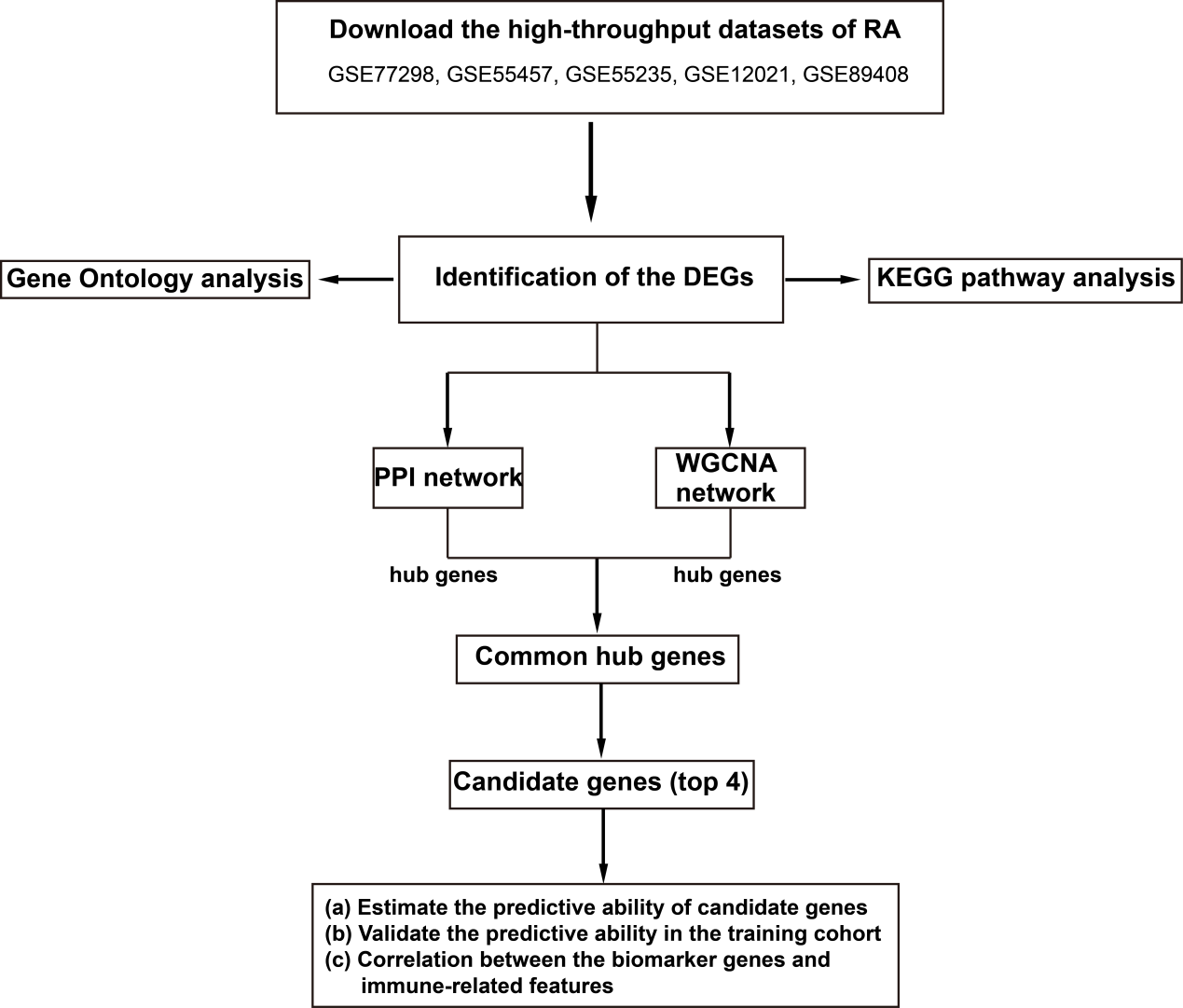
**

**
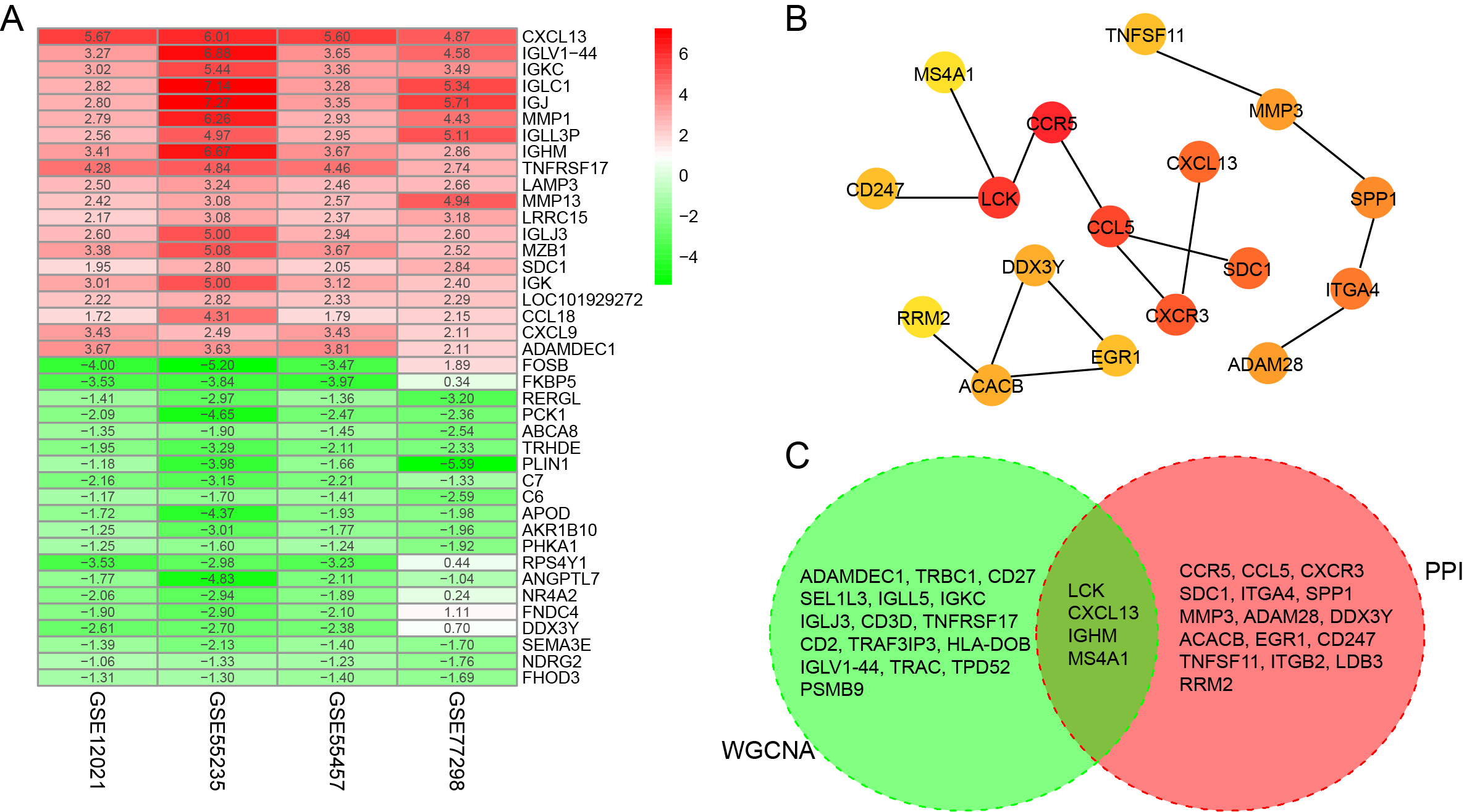
**

**Supplementary Figure S2.** **(A)** Heatmap of the top 20 down-regulated and top 20 up-regulated DEGs in four RA datasets drawn by the “pheatmap” package in R (version 3.6.3) (<http://cran.r-project.org/bin/windows/base/old/3.6.3/>.)

(GSE12021, GSE55235, GSE55457, GSE77298). **(B)** The PPI modules identified using GeNets algorithm. **(C)** Venn diagram of the DEGs in WGCNA and PPI.


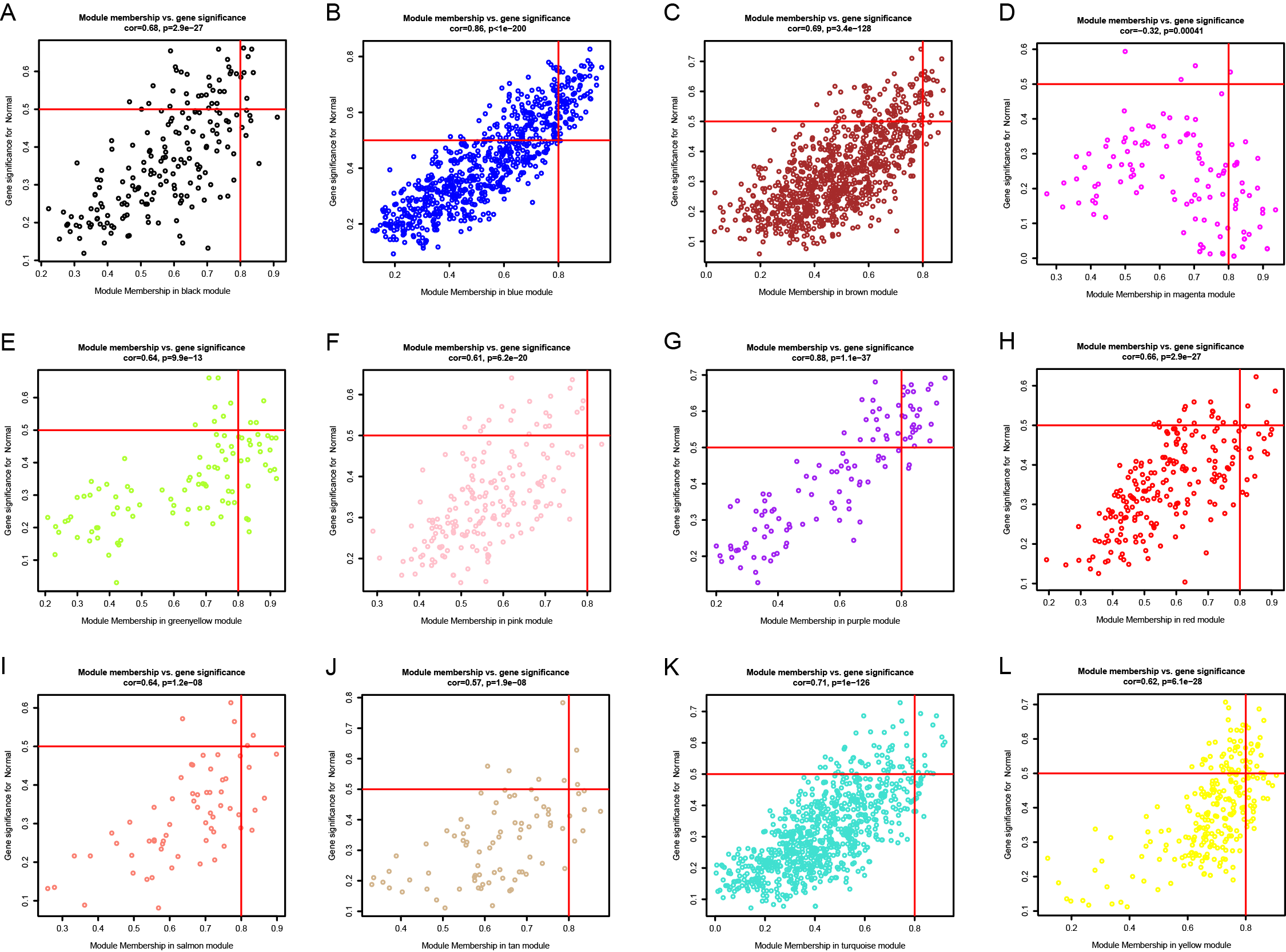


**Supplementary Figure S3. Scatter plot of the gene significance for the normal samples vs. the module membership in the 12 hub modules.**

**
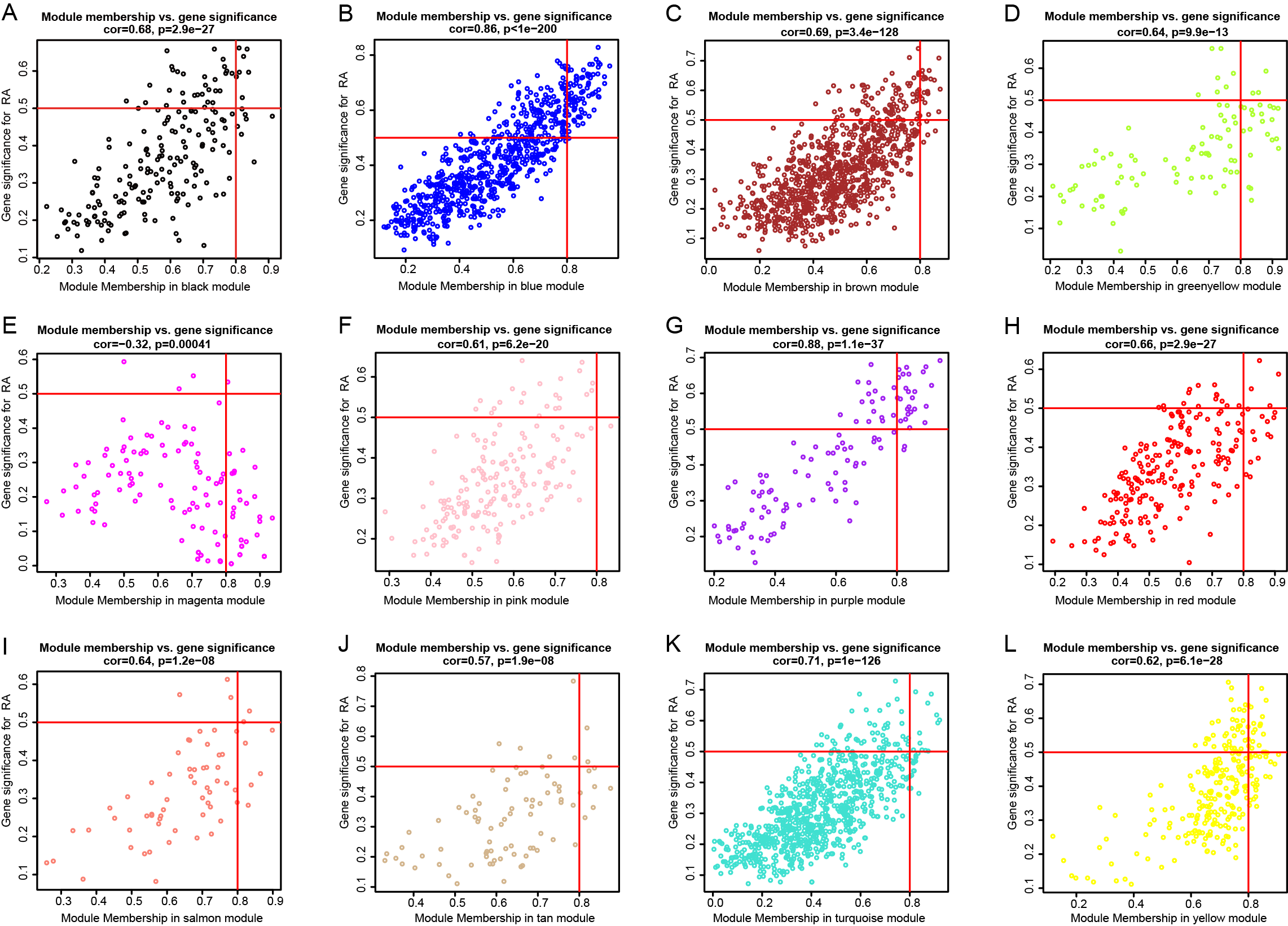
**

**Supplementary Figure S4. Scatter plot of the gene significance for the RA samples vs. the module membership in the 12 hub modules.**

**
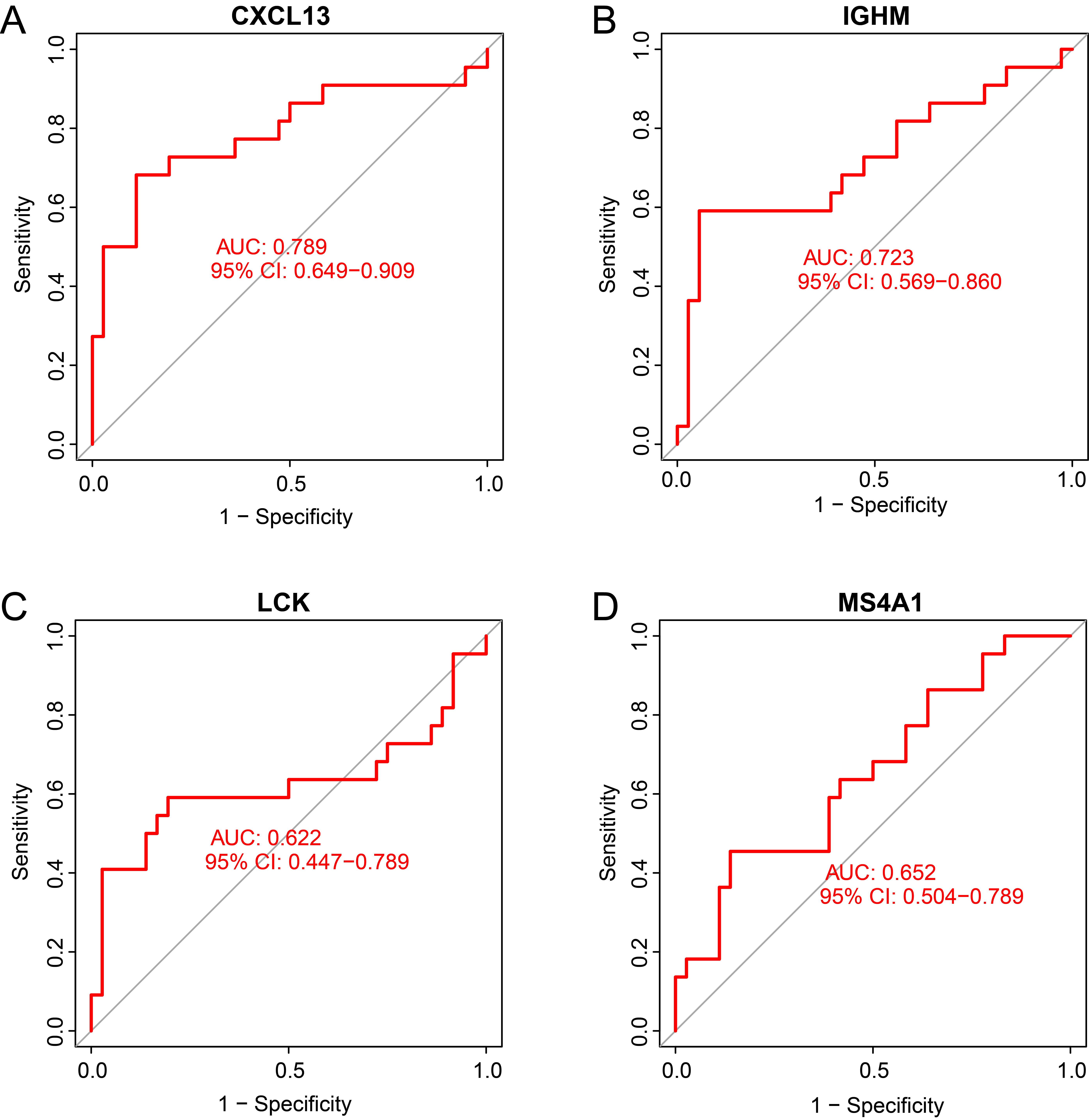
**

**Supplementary Figure S5. ROC curve analysis in the external validation cohort 1 (GSE121894). (A)** ROC curves of the expression of CXCL13 to predict RA. **(B)** ROC curves of the expression of IGHM to predict RA. **(C)** ROC curves of the expression of LCK to predict RA. **(D)** ROC curves of the expression of MS4A1 to predict RA.

**Supplementary Table S1. The significant DEGs between RA and control samples.**

| **Gene Symbol** | **P_value** | **Adjust_P** | **Log_FC** | **Up-/down-regulated** |
| --- | --- | --- | --- | --- |
| FOSB | 1.27E-11 | 2.74E-07 | -2.694912204 | Down-regulated |
| FKBP5 | 8.10E-10 | 1.75E-05 | -2.750692685 | Down-regulated |
| RERGL | 1.25E-09 | 2.71E-05 | -2.233084009 | Down-regulated |
| PCK1 | 2.10E-09 | 4.55E-05 | -2.892710415 | Down-regulated |
| ABCA8 | 3.09E-09 | 6.68E-05 | -1.811092177 | Down-regulated |
| TRHDE | 3.92E-09 | 8.48E-05 | -2.41942385 | Down-regulated |
| PLIN1 | 1.60E-08 | 0.000346039 | -3.050698458 | Down-regulated |
| C7 | 1.68E-08 | 0.000363935 | -2.211952761 | Down-regulated |
| C6 | 1.75E-08 | 0.000379369 | -1.718517782 | Down-regulated |
| APOD | 2.48E-08 | 0.000537299 | -2.499990404 | Down-regulated |
| AKR1B10 | 2.81E-08 | 0.000607675 | -1.997943995 | Down-regulated |
| PHKA1 | 3.36E-08 | 0.000725926 | -1.503826091 | Down-regulated |
| RPS4Y1 | 3.85E-08 | 0.000833382 | -2.325477871 | Down-regulated |
| ANGPTL7 | 3.85E-08 | 0.000833382 | -2.43736147 | Down-regulated |
| NR4A2 | 4.71E-08 | 0.0010179 | -1.662465788 | Down-regulated |
| FNDC4 | 5.18E-08 | 0.00111958 | -1.445684436 | Down-regulated |
| DDX3Y | 1.01E-07 | 0.002186072 | -1.74593125 | Down-regulated |
| SEMA3E | 1.02E-07 | 0.002196147 | -1.656332674 | Down-regulated |
| NDRG2 | 1.35E-07 | 0.002913945 | -1.344211284 | Down-regulated |
| FHOD3 | 1.65E-07 | 0.003558064 | -1.423514913 | Down-regulated |
| ACACB | 2.90E-07 | 0.006279489 | -1.470182817 | Down-regulated |
| FABP4 | 3.10E-07 | 0.006714903 | -2.320878355 | Down-regulated |
| LRP1B | 3.28E-07 | 0.00709472 | -1.158356583 | Down-regulated |
| PDK4 | 3.58E-07 | 0.007735659 | -1.74238119 | Down-regulated |
| CHRDL1 | 3.89E-07 | 0.008419075 | -1.577989469 | Down-regulated |
| LPL | 3.93E-07 | 0.008507579 | -1.423643763 | Down-regulated |
| ADH1B | 4.59E-07 | 0.00992072 | -2.536840495 | Down-regulated |
| EREG | 6.39E-07 | 0.013825044 | -1.373219966 | Down-regulated |
| GPRC5A | 6.39E-07 | 0.013825044 | -1.410168512 | Down-regulated |
| LYVE1 | 6.55E-07 | 0.014156893 | -1.203791606 | Down-regulated |
| JUNB | 7.20E-07 | 0.015573931 | -1.135115484 | Down-regulated |
| MAOA | 7.70E-07 | 0.016649597 | -1.804835002 | Down-regulated |
| EIF1AY | 8.07E-07 | 0.017464282 | -1.764058036 | Down-regulated |
| ENO3 | 9.35E-07 | 0.020214277 | -1.385113077 | Down-regulated |
| NFIL3 | 1.04E-06 | 0.022456021 | -1.406257082 | Down-regulated |
| SLC19A2 | 1.07E-06 | 0.023167081 | -1.686186418 | Down-regulated |
| LAMA2 | 1.09E-06 | 0.023546085 | -1.511900208 | Down-regulated |
| PITX1 | 1.30E-06 | 0.028029136 | -1.461597456 | Down-regulated |
| KCNK3 | 1.31E-06 | 0.028317366 | -1.281191645 | Down-regulated |
| CLUL1 | 1.45E-06 | 0.031433229 | -1.121997273 | Down-regulated |
| EGR1 | 1.48E-06 | 0.032083895 | -1.327323786 | Down-regulated |
| TMOD1 | 1.55E-06 | 0.03361909 | -1.203839042 | Down-regulated |
| LDB3 | 1.57E-06 | 0.033868812 | -1.58072652 | Down-regulated |
| TMEM255A | 1.61E-06 | 0.034881642 | -1.052662322 | Down-regulated |
| FMO2 | 1.69E-06 | 0.036443267 | -1.114001922 | Down-regulated |
| NR4A1 | 1.72E-06 | 0.037243026 | -1.220503677 | Down-regulated |
| TPD52L1 | 1.72E-06 | 0.037243455 | -1.136578671 | Down-regulated |
| ARC | 1.88E-06 | 0.040588737 | -1.109887307 | Down-regulated |
| CXCL13 | 2.37E-14 | 5.12E-10 | 5.537408024 | Up-regulated |
| IGLV1-44 | 7.49E-14 | 1.62E-09 | 4.596558884 | Up-regulated |
| IGKC | 2.68E-13 | 5.79E-09 | 3.826867993 | Up-regulated |
| IGLC1 | 7.02E-13 | 1.52E-08 | 4.643464758 | Up-regulated |
| IGJ | 1.20E-12 | 2.59E-08 | 4.781449841 | Up-regulated |
| MMP1 | 1.53E-12 | 3.30E-08 | 4.101216786 | Up-regulated |
| IGLL3P | 2.92E-12 | 6.33E-08 | 3.898444679 | Up-regulated |
| IGHM | 5.12E-12 | 1.11E-07 | 4.151462707 | Up-regulated |
| TNFRSF17 | 2.17E-11 | 4.69E-07 | 4.078165697 | Up-regulated |
| LAMP3 | 2.74E-11 | 5.93E-07 | 2.715042897 | Up-regulated |
| MMP13 | 3.43E-11 | 7.41E-07 | 3.25370353 | Up-regulated |
| LRRC15 | 3.43E-11 | 7.41E-07 | 2.700808706 | Up-regulated |
| IGLJ3 | 4.23E-11 | 9.15E-07 | 3.284526091 | Up-regulated |
| MZB1 | 8.19E-11 | 1.77E-06 | 3.663196067 | Up-regulated |
| SDC1 | 1.55E-10 | 3.36E-06 | 2.410909518 | Up-regulated |
| IGK | 1.80E-10 | 3.89E-06 | 3.381931254 | Up-regulated |
| LOC101929272 | 3.26E-10 | 7.06E-06 | 2.415864597 | Up-regulated |
| CCL18 | 6.43E-10 | 1.39E-05 | 2.494603047 | Up-regulated |
| CXCL9 | 8.68E-10 | 1.88E-05 | 2.865012832 | Up-regulated |
| ADAMDEC1 | 1.05E-09 | 2.27E-05 | 3.302435868 | Up-regulated |
| SLAMF8 | 1.15E-09 | 2.48E-05 | 2.370756523 | Up-regulated |
| CXCL10 | 1.76E-09 | 3.80E-05 | 2.660954216 | Up-regulated |
| GZMH | 1.76E-09 | 3.80E-05 | 2.186339064 | Up-regulated |
| SLAMF7 | 1.76E-09 | 3.80E-05 | 2.231684384 | Up-regulated |
| NKG7 | 2.10E-09 | 4.55E-05 | 3.261586557 | Up-regulated |
| HLA-DRB4 | 2.14E-09 | 4.63E-05 | 2.339864991 | Up-regulated |
| GUSBP11 | 2.49E-09 | 5.38E-05 | 2.467676904 | Up-regulated |
| CD52 | 4.05E-09 | 8.76E-05 | 2.406670531 | Up-regulated |
| IL21R | 4.18E-09 | 9.05E-05 | 2.38977317 | Up-regulated |
| CCL5 | 4.32E-09 | 9.35E-05 | 2.018089815 | Up-regulated |
| IL7R | 5.22E-09 | 0.000112923 | 1.895450858 | Up-regulated |
| PNOC | 5.55E-09 | 0.000120035 | 2.233883305 | Up-regulated |
| AIM2 | 1.14E-08 | 0.000246399 | 2.284117378 | Up-regulated |
| IGLL5 | 1.26E-08 | 0.000273449 | 2.743990778 | Up-regulated |
| KIF20A | 1.92E-08 | 0.00041505 | 1.996661058 | Up-regulated |
| TRAT1 | 2.01E-08 | 0.000433807 | 2.555790992 | Up-regulated |
| CD2 | 2.38E-08 | 0.000515259 | 1.936980661 | Up-regulated |
| CD72 | 2.43E-08 | 0.000526192 | 2.007709902 | Up-regulated |
| HLA-DOB | 2.47E-08 | 0.000533987 | 2.495519554 | Up-regulated |
| IL32 | 2.47E-08 | 0.000533987 | 2.109065092 | Up-regulated |
| RASGRP1 | 3.98E-08 | 0.000860649 | 2.070880973 | Up-regulated |
| CD3D | 4.44E-08 | 0.000960335 | 1.942685094 | Up-regulated |
| CCR5 | 4.52E-08 | 0.000977756 | 1.995191943 | Up-regulated |
| MMP3 | 4.85E-08 | 0.001049819 | 2.086712754 | Up-regulated |
| ITGA4 | 5.12E-08 | 0.001106426 | 1.679065792 | Up-regulated |
| TNFSF11 | 5.77E-08 | 0.001247396 | 1.670643818 | Up-regulated |
| CD27 | 5.87E-08 | 0.001268583 | 2.046329904 | Up-regulated |
| CCL19 | 6.07E-08 | 0.001311765 | 1.560393331 | Up-regulated |
| TOP2A | 6.48E-08 | 0.001401426 | 1.730317315 | Up-regulated |
| DLGAP5 | 6.69E-08 | 0.001447943 | 1.934121035 | Up-regulated |
| LGALS2 | 6.77E-08 | 0.001464752 | 2.200946938 | Up-regulated |
| IGHD | 6.77E-08 | 0.001464752 | 2.663013747 | Up-regulated |
| TRAF3IP3 | 8.61E-08 | 0.001863014 | 1.536507423 | Up-regulated |
| ITGB7 | 9.86E-08 | 0.002132495 | 1.516946319 | Up-regulated |
| SPAG4 | 1.17E-07 | 0.002536043 | 1.822320851 | Up-regulated |
| FZD2 | 1.22E-07 | 0.002645301 | 1.516660732 | Up-regulated |
| TRBC1 | 1.26E-07 | 0.002715467 | 2.212004492 | Up-regulated |
| PIM2 | 1.33E-07 | 0.002874368 | 1.489701038 | Up-regulated |
| SEL1L3 | 1.46E-07 | 0.003160062 | 1.927513879 | Up-regulated |
| CDC20 | 1.48E-07 | 0.00320255 | 1.836254664 | Up-regulated |
| CEP55 | 1.54E-07 | 0.003332586 | 1.695560045 | Up-regulated |
| HCP5 | 1.58E-07 | 0.003421451 | 1.679860643 | Up-regulated |
| LCK | 1.64E-07 | 0.003545459 | 2.156854568 | Up-regulated |
| RRM2 | 1.82E-07 | 0.003942387 | 1.701969942 | Up-regulated |
| CAPG | 1.92E-07 | 0.004145824 | 1.392374509 | Up-regulated |
| PTPRCAP | 1.97E-07 | 0.004268189 | 1.872461139 | Up-regulated |
| ISG20 | 2.04E-07 | 0.004411074 | 1.821324299 | Up-regulated |
| CORO1A | 2.19E-07 | 0.004745947 | 1.867522614 | Up-regulated |
| SNX10 | 2.27E-07 | 0.004920359 | 1.36672022 | Up-regulated |
| CRTAM | 2.44E-07 | 0.005283559 | 1.929934096 | Up-regulated |
| CYTIP | 2.50E-07 | 0.005408979 | 1.511466636 | Up-regulated |
| DAZL | 2.68E-07 | 0.005798663 | 1.714348309 | Up-regulated |
| IGHG1 | 2.77E-07 | 0.005995248 | 1.880556503 | Up-regulated |
| BLNK | 2.90E-07 | 0.006279489 | 1.467181635 | Up-regulated |
| GZMK | 3.07E-07 | 0.006640808 | 2.126306707 | Up-regulated |
| BCL11B | 3.07E-07 | 0.006640808 | 1.357590983 | Up-regulated |
| MELK | 3.07E-07 | 0.006640808 | 1.449951002 | Up-regulated |
| KIF11 | 3.69E-07 | 0.007986864 | 1.559362794 | Up-regulated |
| PLA2G2D | 3.95E-07 | 0.008534127 | 2.70994599 | Up-regulated |
| PLEKHO1 | 4.02E-07 | 0.00868668 | 1.187398533 | Up-regulated |
| RAC2 | 4.19E-07 | 0.00905335 | 1.717825598 | Up-regulated |
| P2RX5 | 4.54E-07 | 0.009811241 | 1.877450398 | Up-regulated |
| CDK5 | 4.63E-07 | 0.010020802 | 1.389614515 | Up-regulated |
| QPCT | 4.73E-07 | 0.010223236 | 1.455193572 | Up-regulated |
| TLR8 | 4.87E-07 | 0.010532619 | 2.038132791 | Up-regulated |
| SLC2A6 | 4.92E-07 | 0.01063729 | 1.6401481 | Up-regulated |
| CXCL6 | 4.97E-07 | 0.010742739 | 1.969418372 | Up-regulated |
| ITM2C | 5.53E-07 | 0.011955153 | 1.508146175 | Up-regulated |
| IGLV6-57 | 5.53E-07 | 0.011955153 | 1.58452704 | Up-regulated |
| TPX2 | 5.58E-07 | 0.012070244 | 1.243084976 | Up-regulated |
| LCP1 | 5.74E-07 | 0.012420506 | 1.236535895 | Up-regulated |
| HLA-DRB6 | 5.85E-07 | 0.012658212 | 1.250331856 | Up-regulated |
| LOC100293211 | 5.89E-07 | 0.012735003 | 2.166860134 | Up-regulated |
| LY9 | 6.13E-07 | 0.013267405 | 1.222657703 | Up-regulated |
| ASPM | 6.14E-07 | 0.013272571 | 1.903683862 | Up-regulated |
| FKBP11 | 6.31E-07 | 0.013643318 | 1.673964049 | Up-regulated |
| ICAM3 | 6.61E-07 | 0.01428752 | 1.379645353 | Up-regulated |
| TYMS | 6.85E-07 | 0.014819093 | 1.282166438 | Up-regulated |
| RHOH | 7.84E-07 | 0.016945582 | 1.903409584 | Up-regulated |
| SPP1 | 8.40E-07 | 0.018169161 | 2.089000088 | Up-regulated |
| GAP43 | 9.07E-07 | 0.01962361 | 1.099652683 | Up-regulated |
| SEMA4A | 9.15E-07 | 0.019790441 | 1.141379146 | Up-regulated |
| TACC3 | 9.62E-07 | 0.020813876 | 1.402348528 | Up-regulated |
| SAC3D1 | 1.08E-06 | 0.023356006 | 1.210627439 | Up-regulated |
| PVRIG | 1.14E-06 | 0.024711034 | 1.251401867 | Up-regulated |
| IL2RG | 1.17E-06 | 0.025309458 | 1.714521289 | Up-regulated |
| DOCK10 | 1.19E-06 | 0.025714402 | 1.719331989 | Up-regulated |
| AQP9 | 1.26E-06 | 0.02717007 | 2.190035548 | Up-regulated |
| CXCR3 | 1.32E-06 | 0.02846623 | 1.101207038 | Up-regulated |
| SLC39A8 | 1.38E-06 | 0.029808221 | 1.306396152 | Up-regulated |
| NUDT1 | 1.38E-06 | 0.029808221 | 1.314679055 | Up-regulated |
| DHRS9 | 1.39E-06 | 0.030036417 | 1.318928753 | Up-regulated |
| TRAC | 1.39E-06 | 0.030161489 | 1.771108351 | Up-regulated |
| KMO | 1.40E-06 | 0.03026592 | 1.111134959 | Up-regulated |
| LOC102723479 | 1.41E-06 | 0.030496737 | 1.744685012 | Up-regulated |
| SHCBP1 | 1.43E-06 | 0.030962327 | 1.051673354 | Up-regulated |
| CECR1 | 1.45E-06 | 0.031433229 | 1.496310382 | Up-regulated |
| MS4A1 | 1.48E-06 | 0.032083895 | 2.032854377 | Up-regulated |
| CD247 | 1.58E-06 | 0.034086203 | 1.710382308 | Up-regulated |
| GZMB | 1.58E-06 | 0.034086203 | 1.846549958 | Up-regulated |
| GPR18 | 1.59E-06 | 0.034372429 | 1.668802848 | Up-regulated |
| PLXNC1 | 1.61E-06 | 0.034881642 | 1.760537542 | Up-regulated |
| FAM46C | 1.64E-06 | 0.035396491 | 1.086395873 | Up-regulated |
| CST7 | 1.71E-06 | 0.036975277 | 1.314749412 | Up-regulated |
| PRKCZ | 1.72E-06 | 0.037243026 | 1.620428092 | Up-regulated |
| ADAM28 | 1.73E-06 | 0.03751309 | 1.61130643 | Up-regulated |
| CD3G | 1.76E-06 | 0.038056749 | 1.285718388 | Up-regulated |
| KCNN4 | 1.84E-06 | 0.039723224 | 1.367049775 | Up-regulated |
| EVI2B | 1.86E-06 | 0.04029069 | 1.124709983 | Up-regulated |
| CXCR4 | 1.89E-06 | 0.040864213 | 1.283364571 | Up-regulated |
| GZMA | 1.94E-06 | 0.042029608 | 1.421392971 | Up-regulated |
| TK1 | 1.96E-06 | 0.042324811 | 1.18163882 | Up-regulated |
| ROR2 | 2.01E-06 | 0.043521198 | 1.10524172 | Up-regulated |
| GNLY | 2.13E-06 | 0.045989882 | 1.065101276 | Up-regulated |
| CD300A | 2.14E-06 | 0.046305691 | 1.079992122 | Up-regulated |
| ITGB2 | 2.25E-06 | 0.048562131 | 1.293702693 | Up-regulated |

**Supplementary Table S3. The results of KEGG function enrichment analysis.**

| **Pathway names** | **P_value** | **P.adjust** | **Count** |
| --- | --- | --- | --- |
| Cytokine-cytokine receptor interaction | 1.01E-10 | 1.54E-08 | 17 |
| Viral protein interaction with cytokine and cytokine receptor | 3.35E-10 | 2.54E-08 | 11 |
| Chemokine signaling pathway | 3.37E-08 | 1.71E-06 | 12 |
| Rheumatoid arthritis | 7.11E-07 | 2.70E-05 | 8 |
| Hematopoietic cell lineage | 1.15E-06 | 3.50E-05 | 8 |
| Th17 cell differentiation | 2.23E-06 | 5.19E-05 | 8 |
| Intestinal immune network for IgA production | 2.39E-06 | 5.19E-05 | 6 |
| Th1 and Th2 cell differentiation | 8.43E-06 | 0.000160219 | 7 |
| Primary immunodeficiency | 1.22E-05 | 0.000206432 | 5 |
| Cell adhesion molecules | 2.43E-05 | 0.000369458 | 8 |
| Human T-cell leukemia virus 1 infection | 0.000394871 | 0.005456395 | 8 |
| PD-L1 expression and PD-1 checkpoint pathway in cancer | 0.000735518 | 0.009316562 | 5 |
| IL-17 signaling pathway | 0.000942905 | 0.011024741 | 5 |
| Epstein-Barr virus infection | 0.001172205 | 0.012726792 | 7 |
| Viral myocarditis | 0.001363535 | 0.013273847 | 4 |
| Toll-like receptor signaling pathway | 0.001484575 | 0.013273847 | 5 |
| T cell receptor signaling pathway | 0.001484575 | 0.013273847 | 5 |
| Inflammatory bowel disease | 0.00183673 | 0.015510162 | 4 |
| Leukocyte transendothelial migration | 0.002227309 | 0.01781847 | 5 |
| Leishmaniasis | 0.003414518 | 0.024183243 | 4 |
| Axon guidance | 0.00342718 | 0.024183243 | 6 |
| Allograft rejection | 0.003500206 | 0.024183243 | 3 |
| Natural killer cell mediated cytotoxicity | 0.004063888 | 0.026856998 | 5 |
| Graft-versus-host disease | 0.004654808 | 0.029480448 | 3 |
| Type I diabetes mellitus | 0.004975227 | 0.03024938 | 3 |
| ECM-receptor interaction | 0.005506185 | 0.032190005 | 4 |
| Human immunodeficiency virus 1 infection | 0.007175525 | 0.04039555 | 6 |
| Autoimmune thyroid disease | 0.008917277 | 0.048346869 | 3 |
| Chagas disease | 0.009224074 | 0.048346869 | 4 |
| NF-kappa B signaling pathway | 0.009861498 | 0.049964925 | 4 |
